# The Impact of Mental Health and Sociodemographic Characteristics on Quality of Life and Life Satisfaction during the Second Year of the COVID-19 Pandemic—Results of a Population-Based Survey in Germany

**DOI:** 10.3390/ijerph19148734

**Published:** 2022-07-18

**Authors:** Alina Geprägs, David Bürgin, Jörg M. Fegert, Elmar Brähler, Vera Clemens

**Affiliations:** 1 Hospital of Child and Adolescent Psychiatry/Psychotherapy, University of Ulm, 89075 Ulm, Germany; alina.gepraegs@uniklinik-ulm.de (A.G.); david.buergin@upk.ch (D.B.); joerg.fegert@uniklinik-ulm.de (J.M.F.); 2 Child and Adolescent Psychiatric Research Department (UPKKJ), Psychiatric University Hospitals, University of Basel, 4002 Basel, Switzerland; 3 Department for Psychosomatic Medicine and Psychotherapy, University Medical Center of Johannes Gutenberg University of Mainz, 55131 Mainz, Germany; elmar.braehler@medizin.uni-leipzig.de; 4 Integrated Research and Treatment Center Adiposity Diseases, Behavioral Medicine Unit, Department of Psychosomatic Medicine and Psychotherapy, Leipzig University Medical Center, 04103 Leipzig, Germany

**Keywords:** COVID-19, pandemic, quality of life, life satisfaction, mental health, depressive symptoms

## Abstract

A decreased quality of life was shown for numerous factors at the beginning of the pandemic. However, it is important to identify people who are at-risk for long-term impairments during the pandemic and its aftermath. Within this study, we aimed to investigate quality of life within a German population-based sample (2515 participants; 51.6% female; mean age 50.09 years) during the second year of the pandemic (2021). Our results showed that the majority reported no pandemic-associated change in quality of life at this state of the pandemic. Higher life satisfaction was associated with fewer mental health problems, no pre-existing somatic and psychiatric disorders, higher income, no income loss during the pandemic, living with others, and younger age. In contrast, in a high-risk group encompassing participants with lower quality of life, only mental health, pre-existing somatic disorders, and living alone had significant associations with quality of life, indicating a smaller scope for improvement in this high-risk group. Age, income loss, and depressive symptoms predicted a decrease in quality of life since the beginning of the pandemic. Our results highlight the importance of mental health, especially in times of pandemic, and underline the need for low-threshold mental health support.

## 1. Introduction

Since 2019, the novel severe acute respiratory syndrome coronavirus-2 (SARS-CoV-2), and the associated disease COVID-19, has been spreading globally and was declared a pandemic by the WHO shortly after its outbreak [1]. In addition to the many deaths due to the virus, many patients needed intensive care and ventilation, threatening to overload the available resources of healthcare systems. Therefore, many governments enforced various measures to counteract the spreading of the virus, including school closures, contact restrictions, closure of non-essential retail, gastronomy, sports, and leisure time activities [2].

Even though these measures have proven to be effective to reduce COVID-19 cases [3,4], they have various impacts on the population. In addition to loneliness due to reduced social interaction [5] and increased stress, especially for parents working at home while supporting their children with home schooling [6], there are several negative impacts for mental health and quality of life. Compared to results before the pandemic, most studies show significantly higher rates of anxiety and depression (for example, Refs. [7,8,9,10,11]) and reduced quality of life in different countries from all over the world [12,13,14]. Taken together, studies show that measures to reduce the spread of COVID-19 were effective at the price of increased risk for other negative side effects such as decreased quality of life and mental health problems.

Special attention should be drawn towards groups of people that might be more prone to such adverse consequences. As such, studies have shown that certain groups of people have higher risks for depression, anxiety, and lower quality of life during the pandemic, as seen in women [9,12,15,16,17], younger people [9,12,15], people with financial strain [18,19], the unemployed [9,15], and those with previously existing mental health or physical health conditions [12,17,20]. On the other hand, several protective factors were also identified to be unequally distributed in the population, encompassing, among others, living with a partner [9,15,17]. Taken together, studies show several risk and protective factors to be unequally distributed within study samples and populations. Therefore, although most studies use online sample methods to gain large samples, only some reach representativeness [17,19,20]. Population-based and representative samples are thus needed in order to generalize results for the population [21].

While, to date, the majority of studies focus on factors associated to quality of life at the beginning of the pandemic, studies assessing quality of life during later stages of the pandemic are scarce. This is of particular importance as risk of chronicity and mental health consequences are increased with ongoing stress exposure [22]. Considering this, it is important to identify individuals and groups of people who are at risk for low quality of life and to better understand the distribution of risk and protective factors. This will help to provide adequate support for these groups during and after the pandemic and might prevent long-term consequences for patients and future burdens for health care systems. Focusing on protective factors, prevention programs can be developed in order to improve the coping and resiliency of populations in light of the recent and future pandemics. Identifying individuals and groups of individuals who are at risk for adverse outcomes at the population-level is important to adequately target intervention and help efforts.

Quality of life is a subject of which the definition has been controversially discussed [23]. The WHO defined quality of life as the subjective perception of a person of her own position in life in relation to her culture and value system concerning objections, expectations, standards, and concerns [24]. This is a widespread concept summarizing physical, psychological, and ecological aspects. The central component is therefore the subjective evaluation of the person [23]. As described earlier, the pandemic influenced mental health, social contacts, and social life as well as ecological aspects through changes in working conditions. Therefore, changes in quality of life seem likely.

In a recent population-based study, we were able to show that during the second wave of COVID-19 and lockdown in Germany in winter 2021/2022, more than half of participants reported a decreased quality of life compared to before the pandemic. Female gender, age above 60 years, a low household income, not living with a partner, and the experience of childhood adversity were associated with a higher risk for a worsening of quality of life [25].

Within this study, we aimed to investigate life satisfaction and changes in quality of life within a German population-representative sample before and at the beginning of the fourth wave of COVID-19 in Germany in summer/autumn 2021 and to identify factors that affect life satisfaction in particular. Furthermore, we aimed to investigate risk and protective factors in a high-risk group for life satisfaction. To the best of our knowledge, this is the first population-based study focusing on life satisfaction and associated risk and protective factors in the German population above the age of 16 years at later stages of the COVID-19 pandemic. Based on the reviewed literature, we hypothesized that lower life satisfaction is associated with more depressive symptoms, anxiety, and demographic factors such as female gender, younger age, lower income, income loss during the pandemic, living alone, and preexisting mental and physical health conditions.

## 2. Methods

### 2.1. Procedure and Participants

The sample was obtained by a demographic consulting company (USUMA, Berlin, Germany). As the first step, ADM (Arbeitskreis Markt- und Sozialforschungsinstitute e.V.) undertook a systematic area sampling based on the municipal classification of the Federal Republic of Germany, covering the entire inhabited area of Germany. Using these data, around 53,000 areas in Germany were formed electronically, each containing around 700 private households. These 53,000 areas were layered into around 1500 regional layers (according to districts) and then divided into 128 “networks”. These networks are the basis for sampling frames, which contain 258 single sample points. The sample points are proportionate to the distribution of private households in Germany.

In the second selection step, private households at each sample point were systematically selected with a random route procedure. In detail, using a random route approach, households of every third residence in a randomly selected street were invited to participate in the study. In the third step, if there was more than one person meeting the inclusion criteria in the target household (which was asked), the target person was randomly chosen using the Kish selection-grid technique. Participants had to be at least 16 years old and have sufficient German language skills to participate. All selected people were informed about the procedure and the research background and signed informed consent. In an interview conducted after informed consent face-to-face at the participants residence, basic sociodemographic characteristics were assessed by the research staff. In the next step, the research staff handed out a copy of the questionnaire. The participants filled in the questionnaire (paper and pencil) alone with research staff nearby in case of questions. The completed questionnaires were linked to the respondents’ demographic data given in the interview part but did not contain name, address, or any other identifying information. The survey was conducted between July 28 and October 1 2021, thus before and at the beginning of the fourth wave of COVID-19 in Germany. However, while the number of infections reached new highs, there was no lockdown with school closures or shop closures. During the interviews, hygiene measures were taken (wearing mask, keeping distance, disinfecting hands). Of the initial 5934 households contacted, 5908 were occupied at that time. In total, N = 2515 participants were included (utilization rate = 42.6%). The main reasons for non-participation were refusal of the selected household to provide information (24.0%), refusal of the target person to participate (13.6%), and failure to contact people in the household after four attempts (13.4%). The study was conducted in accordance with the Declaration of Helsinki and was approved by the Ethics Committee of the Medical Department of the University of Leipzig.

### 2.2. Measures

*Life satisfaction* was measured using a one item self-rating question (“Currently, how satisfied are you all in all with your life?”) with a scale from 0 (“not satisfied at all”) to 10 (“completely satisfied”) after Beierlein and colleagues [26]. Change in quality of life was measured using the question “Compared to before the COVID-19 pandemic, how would you describe your current quality of life?”. The answer possibilities were “Currently much better than before”, “Currently somewhat better than before”, “Currently about the same”, “Currently somewhat worse than before”, and “Currently much worse than before”. As described before, the answers were summarized in the categories “better”, “equal” and “worse”. [25]. To assess *Depressive symptoms,* the Patient Health Questionnaire-2 (PHQ2) was used. It has a sensitivity of 82% and a specificity of 92% for major depressive disorder [27]. In our sample we saw good internal consistency (Cronbach’s α = 0.82). To assess *Anxiety,* the Generalized Anxiety Disorder 2-item (GAD-2), a screening questionnaire with a sensitivity of 86% and a specificity of 83% for generalized anxiety disorder was used [28]. In our sample we see a satisfying internal consistency (Cronbach’s α = 0.77). *Pre-existing psychiatric disorders and somatic disorders* were assessed using a list of disorders. Participants with any (or several) of these disorders were included in the group with pre-existing psychiatric or somatic disorders. *Loss of income* during the COVID-19 pandemic was separated into two groups (loss of income vs. no loss of income), also living alone. Due to the small number (n = 1) of respondents describing themselves as non-binary/third gender in the sample, these people were excluded from analyses and only the categories “male“ and “female“ were used for the analyses. For income, the equivalent income was used, being calculated from the total income of a household and the number of the people living on this income.

### 2.3. Statistical Analysis

All analyses were performed using SPSS version 28 (IBM Corp, Armonk, NY, U.S.). Comparisons were performed with *t* tests or chi^2^ tests, depending on the included variables. In all regression analyses conducted, life satisfaction was used as the dependent variable. Depressive symptoms, anxiety, pre-existing psychiatric disorders, pre-existing somatic disorders, age, income, living alone, loss of income during COVID-19, and gender were used as independent variables. Predictors were included stepwise into the models. In order to further assess determinants of individuals with high-risk for low life satisfaction, the sample was separated into two groups based on life satisfaction score. Regression analyses were conducted for the group with a life satisfaction ≤ 5. For every linear multiple regression model, homoscedasticity, multicollinearity, normal distribution of residuals, and independence of residuals were checked (see Supplementary Materials). Due to heteroscedasticity, robust standard errors and *p*-values were calculated with a heteroscedasticity-consistent 3 (HC3) procedure in sensitivity analyses. As no differences to the original *p*-values were observed, results of the regression models with non-standardized standard errors were presented. Unadjusted R^2^ values are presented. P-levels are considered as statistically significant at 0.05.

## 3. Results

The final sample included 2515 participants, including 1297 (51.60%) women. The mean age of participants was 50.09 years (*SD* = 18.05). The mean life satisfaction was 7.40 (*SD* = 2.09). The majority of participants reported no change in life quality compared to the time before the pandemic (1461, 62.12%), while about one-third (733, 31.20%) reported a worsening, and only a minority (158, 6.72%) stated their quality of life to be better compared to the time before the pandemic. Detailed sample characteristics are presented in Table 1.

### 3.1. Factors Associated with Life Satisfaction during the Pandemic

To identify factors associated with life satisfaction during the pandemic, linear regression analysis was performed. Step by step, the predictors gender, age, income, living alone, income loss during the COVID-19 pandemic, depressive symptoms, symptoms of anxiety, pre-existing psychiatric disorders, and pre-existing somatic disorders were included in the model. The results for the final model are displayed in Table 2. This model explains a significant and substantial proportion of variance (44.3%) in life satisfaction (R^2^ = 0.443).

Fewer depressive symptoms, fewer anxiety symptoms, no pre-existing psychiatric disorders, no pre-existing somatic disorders, higher income, younger age, not living alone, and no income loss during the pandemic were associated with higher life satisfaction. Gender had no significant influence on life satisfaction (see Table 2).

### 3.2. Factors Associated with Life Satisfaction, Separated in the High-Risk Group with Low Life Satisfaction

For further analyses, a high-risk group for lower life satisfaction was defined (life satisfaction values from 1–5).

In this group, only fewer depressive symptoms, fewer symptoms of anxiety, having a pre-existing somatic disorder, and not living alone were associated with higher life satisfaction. The model explained 17.2% of the variance in life satisfaction (R^2^ = 0.172) (see Table 3).

### 3.3. Factors Associated with Change of Quality of Life Compared to before the Pandemic

Higher age (OR = 1.03, *p* < 0.001) predicted a higher risk in no change in quality of life during the pandemic. Higher age (OR = 1.03, *p* < 0.001), income loss (OR = 2.07, *p* = 0.001), and more depressive symptoms (OR = 3.33, *p* = 0.006) were associated with a higher risk of worse quality of life during the pandemic compared to quality of life being better than before the pandemic (see Table 4).

## 4. Discussion

Our study is, to the best of our knowledge, the first to assess influencing factors on quality of life and life satisfaction during the COVID-19 pandemic within a population-based sample in Germany during this later state of the pandemic. Our results show that most of the German population reported no change in quality of life compared to before the pandemic. Our results show associations between higher life satisfaction with fewer depressive symptoms and symptoms of anxiety as well as no pre-existing somatic or psychiatric disorder, higher income, no income loss during the pandemic, living with others, and younger age. In contrast, in the high-risk group encompassing participants with lower life satisfaction, only mental health, pre-existing somatic disorders, and living alone had significant associations with life satisfaction, while the other predictors showed no significant influence.

In a study conducted in another representative sample of the German population before the pandemic in winter 2017/2018, mean life satisfaction was 8.02 (2.52) [29], while the mean in our sample was 7.40 (2.09). Thus, our data point towards a decrease in life satisfaction in the German population since the beginning of the pandemic. However, in the previous study, the sample was representative for the German population above the age of 14 while in our current study, only participants above the age of 16 were included. Therefore, data may be only partly comparable. Nonetheless, other data also suggest a decrease in life satisfaction in the German population associated with the pandemic, as seen in a survey conducted in winter 2021/2022 with more than half of the participants in a population-based German sample reporting a decreased quality of life since the beginning of the pandemic [25]. In our sample, however, the majority reported about an equal quality of life compared with before the pandemic. This points towards an overall improvement in life quality for the German population compared to earlier stages of the pandemic, which may be due to a relaxation of the situation in Germany. While the aforementioned study took place during lockdown, the current survey was conducted in a time characterized by reopening and easing of contact restrictions.

In line with our hypothesis, fewer depressive symptoms and fewer anxiety symptoms were associated with higher quality of life during the pandemic. This has been shown by studies during the pandemic [30] as well as before the pandemic [31,32]. Having no pre-existing somatic or psychiatric disorder was associated with higher quality of life. This is in line with previous research during the pandemic [12]. In general, it has been shown that people with psychiatric disorders tend to have lower quality of life, independent of the pandemic [33]. As reasons, a lack of coping and adaptation strategies [34] and a higher stress level in general [35] are suggested. Concerning pre-existing somatic disorders, results seem similar. Even before the pandemic, people with pre-existing somatic disorders had lower quality of life [32,36]. Higher income and no income loss during the pandemic were associated with higher quality of life, as we hypothesized. Financial strain has been shown to influence quality of life during [15] as well as before the pandemic [37], with income loss aggravating the situation during the pandemic and therefore having additional impact. During the pandemic, in line with our hypothesis, people who live alone tend to have lower quality of life. This was shown by others [15] and might be due to the contact restrictions, which affected people living alone more. In addition, not being married, often going along with living alone, was already a well-known predictor of lower quality of life before the pandemic [38].

Against our hypothesis, which expected women to have a lower quality of life, gender had no significant impact on quality of life in our sample. This contrasts with several studies [9,12,15,16,17] showing women at higher risk for mental health problems and lower quality of life during the pandemic. Additionally, before the pandemic, women tended to have lower values for quality of life [32]. These differences can be partly explained by the general disadvantage of women in socioeconomic dimensions such as income or marital status [39]. A reason for the pandemic-associated lower quality of life in women could be that in earlier stages of the pandemic, schools and childcare institutions were closed. Mainly women took care of the children [40]. However, our study was conducted at a later stage of the pandemic. Schools and day care for children had re-opened, and the economic crisis of the first lockdown in Germany was largely over [41]. Thus, the situation of women, having been affected in particular by these developments, may have improved [42]. However, more research is needed at this and later stages of the pandemic to strengthen and replicate these findings.

In contrast to our hypothesis and several studies conducted during the pandemic [12,15], there was a negative relationship between age and quality of life, meaning older people tended to have lower quality of life. One explanation could be that in this late stage of the pandemic when our survey was conducted, contact restrictions were less strict. Younger people began to have more social contacts again with a positive effect on quality of life [43], while older people may have tended to be more careful due to the higher risk for severe disease. Thus, at this stage of the pandemic, older people may have been more strongly affected. However, before the pandemic, the literature showed lower quality of life associated with higher age [32,44], more consistent with our findings.

A significant and substantial proportion of variance could be explained in life satisfaction using the risk and protective factors included in our model. Focusing on participants with higher life satisfaction, the predicting factors were similar to the factors within the total sample. Strikingly, in the high-risk group with lower life satisfaction, the majority of assessed factors were not associated significantly with life satisfaction. In detail, only fewer depressive symptoms, fewer anxiety symptoms, having pre-existing somatic disorder, and not living alone were associated with higher life satisfaction. Age, gender, income, income loss, and pre-existing mental health disorder showed no significant impact on life satisfaction in this high-risk group. This is of major importance, implicating these factors as of lower relevance in people who have a lower life satisfaction. Consequently, there may be a smaller scope for improvement in the lower life satisfaction group, being particularly significant as improvement of life satisfaction is of major relevance for this part of society.

### Strengths and Limitations

One of the strengths of this study is our large-scale, population-based sample and thus the possibility to generalize our results to the German population. In addition, we have included different independent variables containing various demographic factors, depressive symptoms, and symptoms of anxiety. However, there are several limitations apparent. First, only data from self-reported screening tools were available and analyzed. These measures were taken from validated short questionnaires implemented in different population-based surveys. Many of these measures are short and self-reported. As such, life satisfaction was assessed by one self-rated question [26]. There are many different questionnaires, partly with up to 100 questions (for example WHOQOL [24]), trying to represent the concept adequately but not efficiently. As described in the introduction, the subjective evaluation of the person is the central component, therefore enabling use in more economical ways. Therefore, single-item questionnaires seem to be equal but more efficient [45]. Last, the study uses a cross-sectional design, and thus no causality can be inferred, and replication of our findings within longitudinal designs would help to elucidate directionality of findings. In addition to these limitations, this study elucidates risk and protective factors for quality of life during a later stage of the COVID-19 pandemic using data from a large population-representative survey of the German population.

## 5. Conclusions

Our results show that most of the German population are not at risk for low life satisfaction. Remarkably, compared to other studies conducted in earlier stages of the pandemic, the majority reported no change in quality of life compared to before the pandemic. However, a certain subgroup of the population reported a lower life satisfaction. In this high-risk group, fewer sociodemographic factors had an influence on life satisfaction, suggesting that potential protective factors have no or less impact on life satisfaction in this group. Depressive symptoms and symptoms of anxiety, on the other hand, still affected life satisfaction significantly, even in this high-risk group. Hence, it is pivotal to focus on mental health in order to increase quality of life. This leads to the possible conclusion that during the course of the pandemic and potentially beyond it, low-threshold support services for mental health and psychotherapy are of major importance. Sufficient resources are required to meet the mental health needs of the population. In particular, programs that counteract the mental health crises in children are urgently needed [46,47]. In addition to mental health, economic support is associated with quality of life. However, economic factors were shown to be of relevance only in the part of the sample already having a relatively high quality of life. In addition to these influenceable factors, demographic factors showed to be significant predictors for quality of life at this state of the pandemic.

In future research, additional factors such as personality structure and functioning should be included to explain more variance in life satisfaction. Our results point towards a decrease in the burden women faced in the second year of COVID-19 compared to the beginning of the pandemic, a finding in need of further research. Longitudinal studies could explore the differences between the respective stages of the pandemic adequately.

## Figures and Tables

**Table 1:** Sample characteristics.

Variable	*N*	*M*/ *N*	*SD*/ %	Range
**Gender**	2514			
Female		1297	51.60%	
Male		1217	48.40%	
**Age**	2515	50.09	18.05	16–101
**Income in Euro**	2470	2015.05	1012.12	125–7500
**Living alone**				
Yes	2515	995	39.60%	-
**Loss of income**				
Yes	2471	483	19.50%	-
**Depressive symptoms**	2514	0.83	1.20	0–6
**Symptoms of anxiety**	2512	0.78	1.12	0–6
**Pre-existing psychiatric disorder**				
Yes	2456	413	16.80%	-
**Pre-existing somatic disorder**				
Yes	2502	928	37.10%	-
**Life satisfaction**	2505	7.40	2.09	0–10
**Compared to before the pandemic, quality of life today is…**				
Better		158	6.72%	
Equal		1461	62.12%	
Worse		733	31.16%	

(*N* = 2456–2515).

**Table 2:** Associations of sociodemographic characteristics and mental health with life satisfaction.

Predictor	*β*	*SE*	*p*
Intercept	8.87 ***	0.12	<0.001
Gender	−0.11	0.07	0.08
Age	−0.01 ***	0.002	<0.001
Income	0.23 ***	0.03	<0.001
Living alone	0.40 ***	0.07	<0.001
Income loss	−0.48 ***	0.08	<0.001
Depression	−0.70 ***	0.04	<0.001
Anxiety	−0.22 ***	0.04	<0.001
Pre-existing psychiatric disorder	−0.61 ***	0.10	<0.001
Pre-existing somatic disorder	−0.36 ***	0.08	<0.001

Presented as beta coefficients (*β*) and standard error (*SE*). (R^2^ = 0.443). (n = 2350). F(9,2349) = 206.85, *p* < 0.001. *** *p* < 0.001.

**Table 3:** Associations of sociodemographic characteristics and mental health with life satisfaction in the high-risk group of participants with lower life satisfaction.

Predictor	*β*	*SE*	*p*
Intercept	4.31 ***	0.30	<0.001
Gender	−0.21	0.13	0.10
Age	−0.003	0.004	0.51
Income	0.13	0.10	0.15
Living alone	0.46 ***	0.14	<0.001
Income loss	−0.23	0.15	0.12
Depression	−0.17 **	0.06	<0.01
Anxiety	−0.21 ***	0.06	<0.001
Pre-existing psychiatric disorder	0.11	0.14	0.43
Pre-existing somatic disorder	0.34 *	0.15	0.02

Presented as beta coefficients (*β*) and standard error (*SE*). (R^2^ = 0.172) (n = 396). F(9,395) = 8.88, *p* < 0.001. *** *p* < 0.001, ** *p* < 0.01, * *p* < 0.05.

**Table 4:** Factors associated with equally and worse-rated quality of life compared to improvements since the beginning of the pandemic.

Predictor	Odds Ratio ^1^	95% CI	*p*
**Quality of life is equal to before the pandemic**			
Gender	1.01	0.72–1.41	0.96
Age	1.03 ***	1.02–1.04	<0.001
Income	0.88	0.75–1.02	0.08
Living alone	1.27	0.88–1.84	0.20
Income loss	0.75	0.49–1.15	0.18
Depressive symptoms	0.99	0.42–2.38	0.99
Symptoms of anxiety	0.49	0.23–1.03	0.06
Pre-existing psychiatric disorder	0.57	0.37–0.90	0.02
Pre-existing somatic disorder	0.97	0.63–1.48	0.89
**Quality of life is worse than before the pandemic**			
Gender	1.09	0.77–1.56	0.62
Age	1.03 ***	1.01–1.04	<0.001
Income	0.82	0.70–0.96	0.02
Living alone	1.15	0.78–1.69	0.48
Income loss	2.07 **	1.34–3.18	0.001
Depressive symptoms	3.33 **	1.41–7.86	0.006
Symptoms of anxiety	0.68	0.32–1.43	0.31
Pre-existing psychiatric disorder	0.90	0.57–1.43	0.66
Pre-existing somatic disorder	1.22	0.79–1.90	0.37

^1^ An OR > 1 corresponds to a higher probability of reporting that quality of life is equal or worse compared to being better than before the pandemic. *** *p* < 0.001, ** *p* < 0.01.

## Data Availability

The data presented in this study are available on request from the corresponding author.

## Supplementary Materials

The following supporting information can be downloaded at: https://www.mdpi.com/article/10.3390/ijerph19148734/s1, Table S1. Multicollinearity statistics for the final regression model on life satisfaction in the total sample (refers to Table 2). Figure S1. Distribution of residuals from the final regression model (life satisfaction). Figure S2. Scatterplot of residuals for the final regression model (life satisfaction). Table S2. Multicollinearity statistics for the final regression model on life satisfaction in the group with lower life satisfaction (refers to Table 3). Figure S3. Distribution of residuals from the final regression model of life satisfaction in the group with lower life satisfaction. Figure S4. Scatterplot of residuals of the final regression model in the group with lower life satisfaction.
