# Supplementary material for: The Impact of Mental Health and Sociodemographic Characteristics on Quality of Life and Life Satisfaction during the Second Year of the COVID-19 Pandemic—Results of a Population-Based Survey in Germany
Source: Int J Environ Res Public Health. 2022 Jul 18;19(14):8734. doi: 10.3390/ijerph19148734 (PMC9316196; doi:10.3390/ijerph19148734)

Annex 1

Table S1

Multicollinearity statistics for the final regression model (see Table 2)

Variable	Variance Inflation Factor (VIF)
Gender	1.03
Age	1.35
Income	1.07
Living alone	1.10
Income loss	1.05
Depression	2.16
Anxiety	2.12
Pre-existing somatic disorder	1.40
Pre-existing psychiatric disorder	1.25

Variance Inflation factors above 10 represent strong evidence for multicollinearity. Durbin Watson statistic=0.81(values below 0.7 and above 2.3 indicate autocorrelation of residuals).

Figure S1

Distribution of residuals from the final regression model (M=0, SD=1, N=2350)

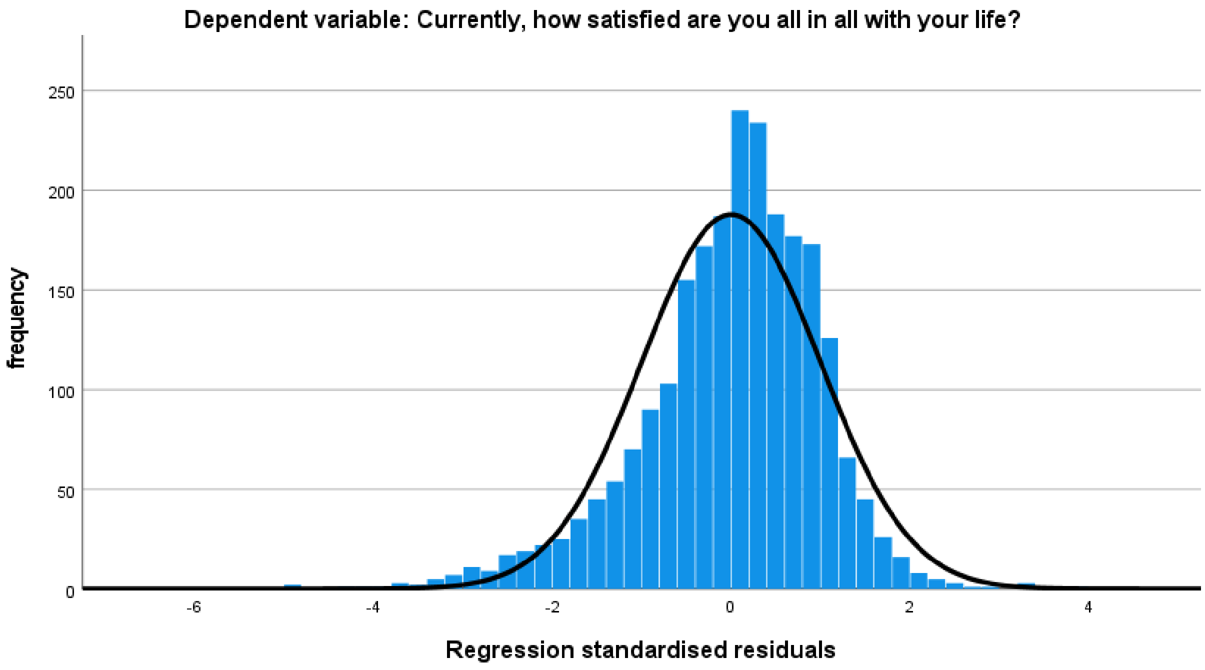

Figure S2

Scatterplot of residuals for the final regression model

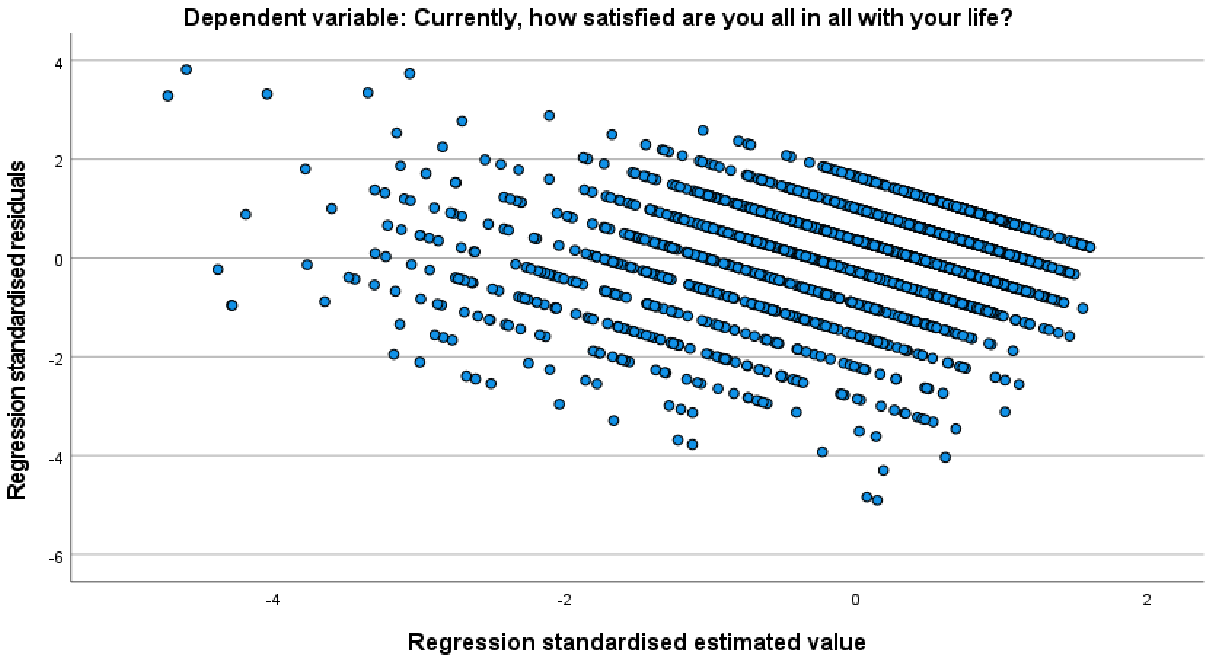

Table S2

Multicollinearity statistics for the final regression model in the group with low quality of life (see Table 3)

Variable	Variance Inflation Factor (VIF)
Gender	1.07
Age	1.50
Income	1.10
Living alone	1.20
Income loss	1.13
Depression	1.86
Anxiety	2.01
Pre-existing somatic disorder	1.28
Pre-existing psychiatric disorder	1.20

Variance Inflation factors above 10 represent strong evidence for multicollinearity. Durbin Watson statistic=0.81(values below 0.7 and above 2.3 indicate autocorrelation of residuals).

Figure S3

Distribution of residuals from the final regression model in the group with low quality of life (M=0, SD=1, N=396)

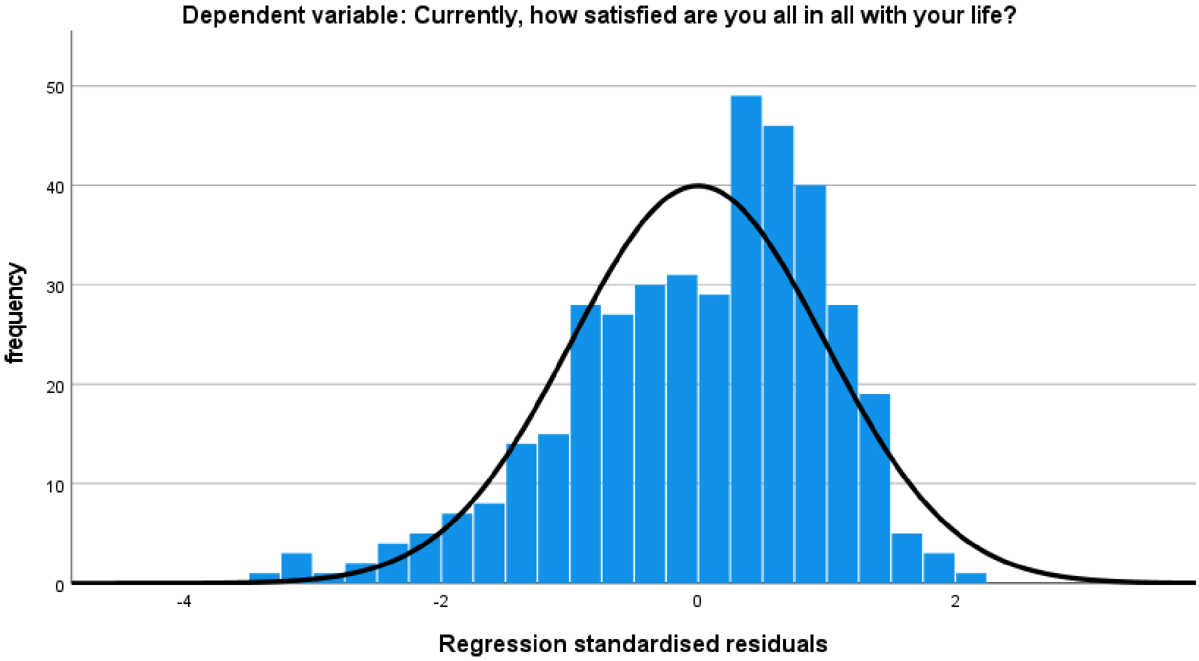

Figure S4

Scatterplot of residuals of the final regression model in the group with low quality of life

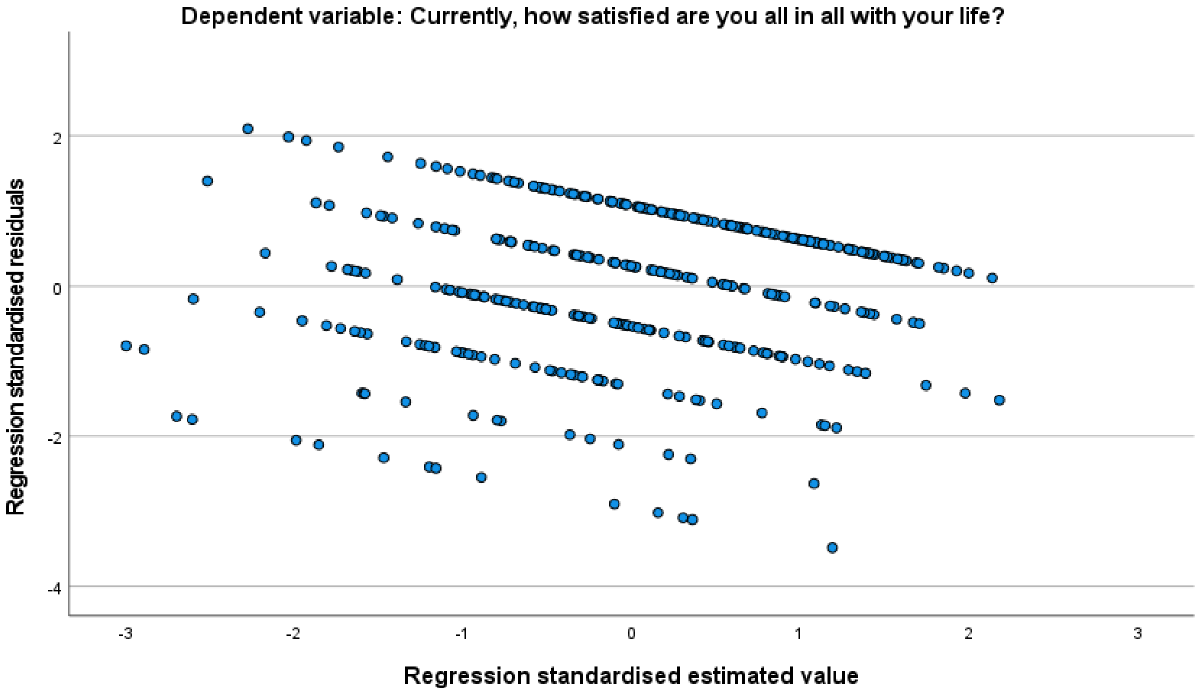

Supplement: Supplementary file 1 [file ijerph-19-08734-s001.zip › ijerph-1797236-supplementary.pdf]
